# Effect of the BOPPPS model combined with case-based learning versus lecture-based learning on ophthalmology education for five-year paediatric undergraduates in Southwest China

**DOI:** 10.1186/s12909-022-03514-4

**Published:** 2022-06-07

**Authors:** Lin Chen, Xiao-Jiao Tang, Xin-Ke Chen, Ning Ke, Qin Liu

**Affiliations:** Department of Ophthalmology, Children’s Hospital of Chongqing Medical University, Ministry of Education Key Laboratory of Child Development and Disorders, China International Science and Technology Cooperation Base of Child Development and Critical Disorders, 136, Zhongshan 2nd RD, Yuzhong District, 400014 Chongqing, China

**Keywords:** BOPPPS model, Case-based learning, Undergraduate, Ophthalmology teaching

## Abstract

**Background:**

To investigate the effect of the bridge-in, objective, preassessment, participatory learning, post assessment, and summary (BOPPPS) model combined with case-based learning (CBL) on ophthalmology teaching for five-year paediatric undergraduates.

**Methods:**

The effects of the BOPPPS model combined with CBL (BOPPPS-CBL) and traditional lecture-based learning (LBL) on ophthalmology teaching were compared among students in a five-year programme. The questionnaire surveys of the students were collected and statistically analysed after the class. The final examination scores, including on elementary knowledge and case analysis, in the two groups were analysed.

**Results:**

There were no statistically significant differences between the teachers and students in the baseline data. More students agreed that the BOPPPS-CBL model helped develop their problem-solving skills, analytical skills and motivation for learning better than the LBL model. There was no significant difference in learning pressure between the two groups. The final examination scores of the BOPPPS-CBL group were significantly higher than those of the LBL group. The overall course satisfaction of the BOPPPS-CBL group was obviously higher than that of the LBL group.

**Conclusions:**

The BOPPPS-CBL model is an effective ophthalmology teaching method for five-year paediatric undergraduates.

## Background

Ophthalmology is a highly practical subject that greatly emphasizes the clinical thinking ability and complex clinical problem solving. Ophthalmic education is a cornerstone to improving eye health care globally [1]. It is essential not only for the training of future ophthalmologists but also for medical practitioners from other disciplines in general, as visual system dysfunction may provide clues for the diagnosis of systemic diseases [2, 3]. However, the decline in ophthalmology experience and exposure in medical schools was universal and severe [1, 4]. It is essential to propose new educational responses and reforms to mitigate the decline in exposure and training in ophthalmic education. Ophthalmology education is a unit of sensory system teaching for five-year programme students at the College of Pediatrics of Chongqing Medical University. Limited teaching time is also a dilemma for our ophthalmic education for undergraduates. Traditional lecture-based learning (LBL) instruction has been the foundation for transferring basic information to medical students on clinical rotations, which is typically focused on the teacher and how facts are transferred to the student for retention and later recall [5]. Little attention is given to problem solving, which may inhibit the learning initiative and enthusiasm of students. Therefore, strategies that improve learning initiative and self-learning ability are the focus of educational innovation in China.

The bridge-in, objective, preassessment, participatory learning, post assessment, and summary (BOPPPS) model originated in the Instructional Skill Workshop (ISW) in British Columbia, Canada, in the 1970s [6]. Based on constructivism and communication methods, the BOPPPS teaching model constructs a complete teaching process and theoretical framework for the achievement of teaching objectives, forms a closed loop teaching unit with a complete system, and pays more attention to the effectiveness of teaching objectives and the diversity of teaching methods [7]. The BOPPPS model divides the classroom teaching process into 6 stages (or elements): bridge-in (B), objective (O), preassessment (P), participatory learning (P), postassessment (P) and summary (S). In recent years, this model has been widely considered by Chinese educational institutions and colleges and has been gradually applied to medical teaching [7, 8].

Ophthalmic education has many knowledge points and focuses on the ability to combine theory with practice [9]. Diminishing ophthalmology clerkships and limited teaching time devoted to ophthalmology in medical curricula globally is a recurrent issue [1, 4]. A consensus has emerged that strategies are needed to focus on how to optimize the limited time allotted to ophthalmology [4]. For decades traditional lecture-based learning (LBL) has been the most widely used model in China [10]. LBL centres teaching around lecture-based instruction, emphasizing the delivery of syllabuses and concepts [11]. In traditional LBL teaching in China, teachers are mainly active, while students play a more passive role in accepting knowledge. LBL often leads to unsatisfactory learning outcomes because medical students passively receive knowledge from instructors with little interaction, and lack motivation to study and innovate [12, 13]. Therefore, in recent years, educators have been innovating teaching models (problem-based lecture, team-based learning, flipped classroom) to improve students’ learning interests, innovation ability and so on. These education models show some advantages comparing with traditional LBL [14–16].Case-based learning (CBL) originated from Harvard University in the twentieth century and has been proven to enhance students’ learning by aiding them in linking theory to clinical practice [17]. To improve students’ abilities to analyse and solve problems, teaching strategies increasingly emphasize their active participation. The BOPPPS teaching model has been shown to be more effective than the LBL model in the teaching of other medical specialties [7, 8]. This study was a preliminary investigation into the effectiveness of the BOPPPS-CBL model on ophthalmology education for five-year paediatric undergraduates.

## Methods

### Ethical approval

This study was performed in accordance with the Helsinki Declaration. This research comes from education reform project (the Project on University First-Class Undergraduate Course of Chongqing Education Commission NO.2021–168) and are exempt from Ethics Committee of the Children’s Hospital of Chongqing Medical University. The informed consent was obtained from all participants.

### Participants

The research is a non-randomized control trial. The participants comprised 87 five-year paediatric learning ophthalmology at the Children’s Hospital of Chongqing Medical University from April 1^st^, 2020, to May 30^th^, 2021. All undergraduates studied ophthalmology in their sixth semester. Forty-three undergraduates in 2017 were included in the traditional LBL group (LBL group), and 44 undergraduates in 2018 were included in the BOPPPS-CBL group (BOPPPS-CBL group) (Table 1).

**Table 1:** Characteristics of the participants of the two groups

Characteristics	BOPPPS-CBL group (*n* = 44)	LBL group (*n* = 43)	X^2^/t	*P* Value
Sex				
Male, n (%)	25 (56.82)	21 (48.84)	0.556	0.46
Female, n (%)	19 (43.18)	22 (51.16)		
Age in years, (mean ± SD)	21.39 ± 0.12	21.15 ± 0.14	1.27	0.21
Interest in specialty				
strong	25	26	0.61	0.74
normal	18	15		
lack	1	2		

### Design

**Fig. 1:**
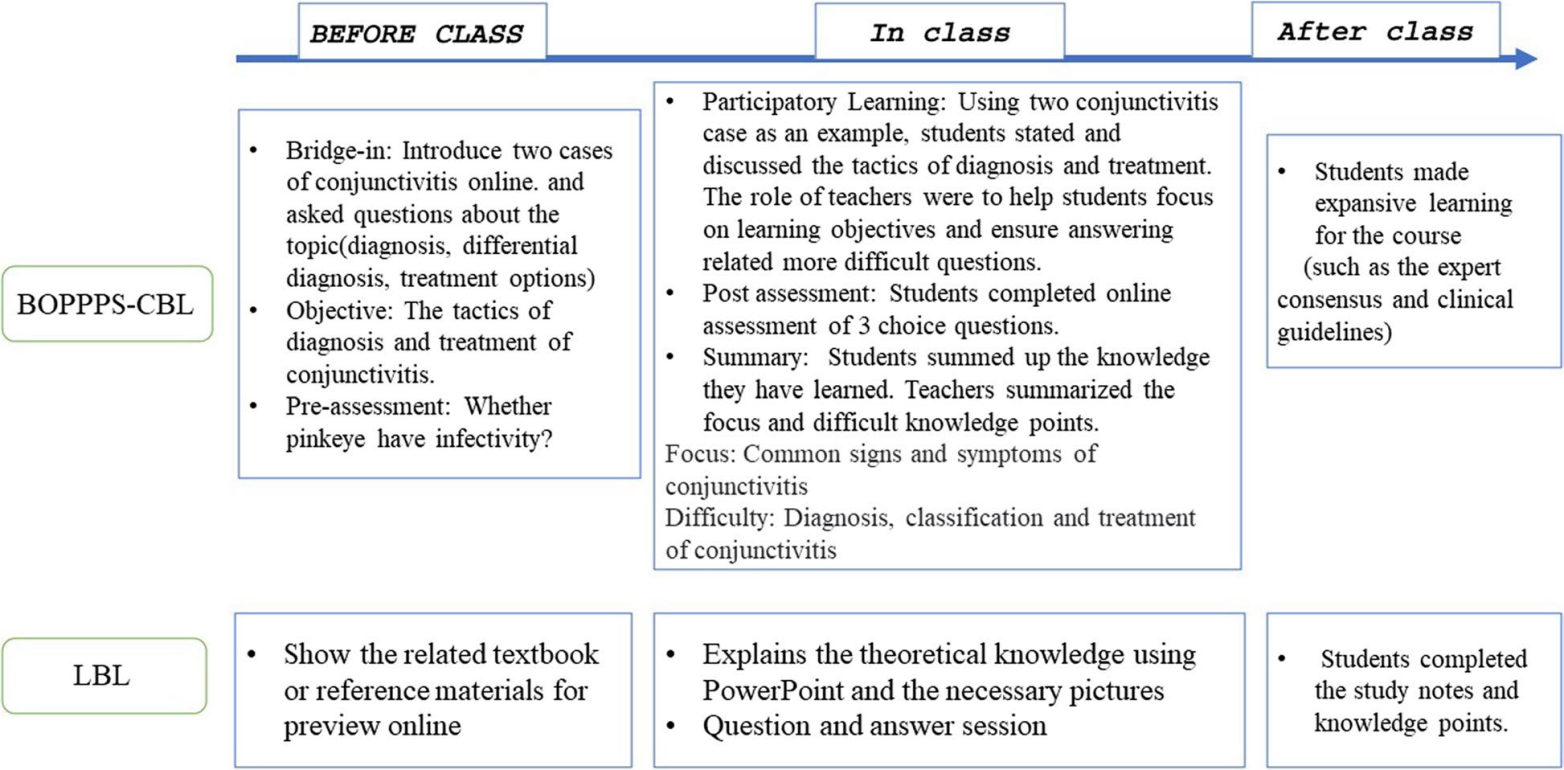
Flowchart of teaching design of the BOPPPS-CBL and LBL groups

The educational material was the Diseases of the Sensory System textbook (Hu GH, Zhou SB. 1st Ed. People’s Medical Publishing House). The BOPPPS-CBL and LBL model flowchart is summarized in Fig. 1. We chose conjunctivitis as the focus to apply the teaching approaches in this study. Briefly, the traditional LBL design included three parts. First, the participants were encouraged to preview the related textbook or reference materials prior to the class. Then, in the class, the teacher explained the theoretical knowledge using PowerPoint and the necessary pictures. The students listened and took notes. Last, the teacher assigned homework to the students. If the students had any questions, the teacher answered them and reviewed the relevant knowledge points after class.

The BOPPPS-CBL model comprises six parts, bridge-in, objective, preassessment, participatory learning, post assessment, and summary, based on the cases. The main purpose of bridge-in design is to enable medical students to understand the main content of the course in a framework and stimulate their strong interest in learning. Before class, the teachers introduced two cases of conjunctivitis online teaching platforms (http://cqmu.fy.chaoxing.com/portal) and motivated the students’ interest in learning clinical diseases characterized by “red eye” and “increased secretions”. According to the course syllabus of the College of Pediatrics of Chongqing Medical University, the objective part clearly specified tactics for the diagnosis and treatment of conjunctivitis as the focus of this course. The preassessment was offered online with the question “Is pinkeye infectious?” In class, based on the two conjunctivitis cases presented online, the students stated and discussed the tactics for diagnosis and treatment. The role of the teachers was to help the students focus on the learning objectives and ensure answers related to more difficult questions. Postassessment was designed with 3 choice questions online. According to the post-assessment results, the teachers adjusted the follow-up teaching content to improve the teaching efficiency. Finally, in class, the students summarized the knowledge they learned, and the teachers summarized the focus and difficult knowledge points. After class, the teachers encouraged the students to learn expansively for the course (such as expert consensus and clinical guidelines).

### Evaluation

After the class, the students from both groups were asked to complete an anonymous questionnaire. The questionnaire was a modified version of the course evaluation questionnaire (CEQ), which has verified reliability and validity [18]. The students’ perspectives and self-evaluations were quantified using a five-point Likert-type scale ranging from a score of one for strong disagreement to a score of five for strong agreement (Table 2). To evaluate the students’ understanding of the course material, we analysed the scores of their fundamental knowledge and case analyses with a final exam at the end of the semester.

**Table 2:** Comparison of the modified course experience questionnaire between the CBL- BOPPPS group and the LBL group

Question	CBL- BOPPPS	LBL	t	*P* Value
It is always easy to know the standard of work expected	4.36 ± 0.87	3.86 ± 1.01	6.33	0.00
The course developed my problem-solving skills	4.34 ± 0.86	3.70 ± 1.12	8.42	0.00
The course sharpened my analytic skills	4.32 ± 0.88	3.79 ± 1.01	6.63	0.00
The course promotes the memorization of knowledge	4.23 ± 0.99	3.70 ± 1.06	4.37	0.00
The course is overly theoretical and abstract	3.50 ± 1.36	3.42 ± 1.05	0.39	0.70
The course helps enhance my motivation to learn	4.25 ± 0.89	3.63 ± 1.05	5.44	0.00
The course gives me too much preclass work	2.52 ± 1.42	2.93 ± 1.01	2.12	0.04
I feel confident about tackling unfamiliar problems	4.20 ± 0.98	3.53 ± 1.12	5.25	0.00
There are many pressure on me to do well in this course	3.07 ± 1.37	3.26 ± 1.23	1.46	0.15
Overall, I am satisfied with the course	4.59 ± 0.69	3.84 ± 1.02	5.96	0.00

This survey adopted a five-point Likert-type scale (1, strongly disagree; 2, disagree; 3, neutral; 4, agree; 5, strongly agree). Values are means ± SD.

### Statistical analysis

All statistical analyses were carried out using SPSS 22.0 (SPSS, Inc., Chicago, IL). The measurement data are expressed as the means ± SD and analysed by the t test. Categorical data were analysed by the chi-square test. Statistical significance was defined as *p* < 0.05.

## Results

The general characteristics of the two groups are shown in Table 1. The five-year paediatric undergraduate students in 2017 (the LBL group) comprised 43 individuals, including 22 females and 21 males. The mean age of the LBL group was 21.15 years. The five-year paediatric undergraduate group in 2018 (the BOPPPS-CBL group) comprised 40 individuals, of whom 19 were female and 25 were male. The mean age of the BOPPPS-CBL group was 21.39 years. Students’ interest in ophthalmology specialty was graded by strong, normal, and lack. No significant differences in general characteristics, including sex, age, and interest in specialty, were found between the two groups (*P* > 0.05).

Table 2 compares the students’ perspectives on the traditional LBL model and the BOPPPS model combined with those on the CBL model. More students agreed that the BOPPPS-CBL model helped develop their problem-solving skills (4.34 ± 0.86 vs. 3.70 ± 1.12, *P* < 0.01), analytical skills (4.32 ± 0.88 vs. 3.79 ± 1.01, *P* < 0.01), and motivation for learning (4.25 ± 0.89 vs. 3.63 ± 1.05, *P* < 0.01) better than the LBL model. More students in the BOPPPS-CBL group (4.36 ± 0.87 vs. 3.86 ± 1.01, *P* < 0.01) than in the LBL group agreed that they knew the expected standard of work. Although students in both groups considered the course to be overly theoretical and abstract, they thought the BOPPPS-CBL model better promoted knowledge retention than the LBL model (4.23 ± 0.99 vs. 3.70 ± 1.06, *P* < 0.01). There was no significant difference in learning pressure between the two groups; however, more students in the LBL group thought the preclass workload was too high (2.93 ± 1.01 vs. 2.52 ± 1.42, *P* = 0.04). Through the BOPPPS-CBL course, more students felt confident about tackling unfamiliar problems than through the LBL course (4.20 ± 0.98 vs. 3.53 ± 1.12, *P* < 0.01). Overall, the course satisfaction of the BOPPPS-CBL group was obviously higher than that of the LBL group (4.59 ± 0.69 vs. 3.84 ± 1.02, *p* < 0.01).

**Fig. 2:**
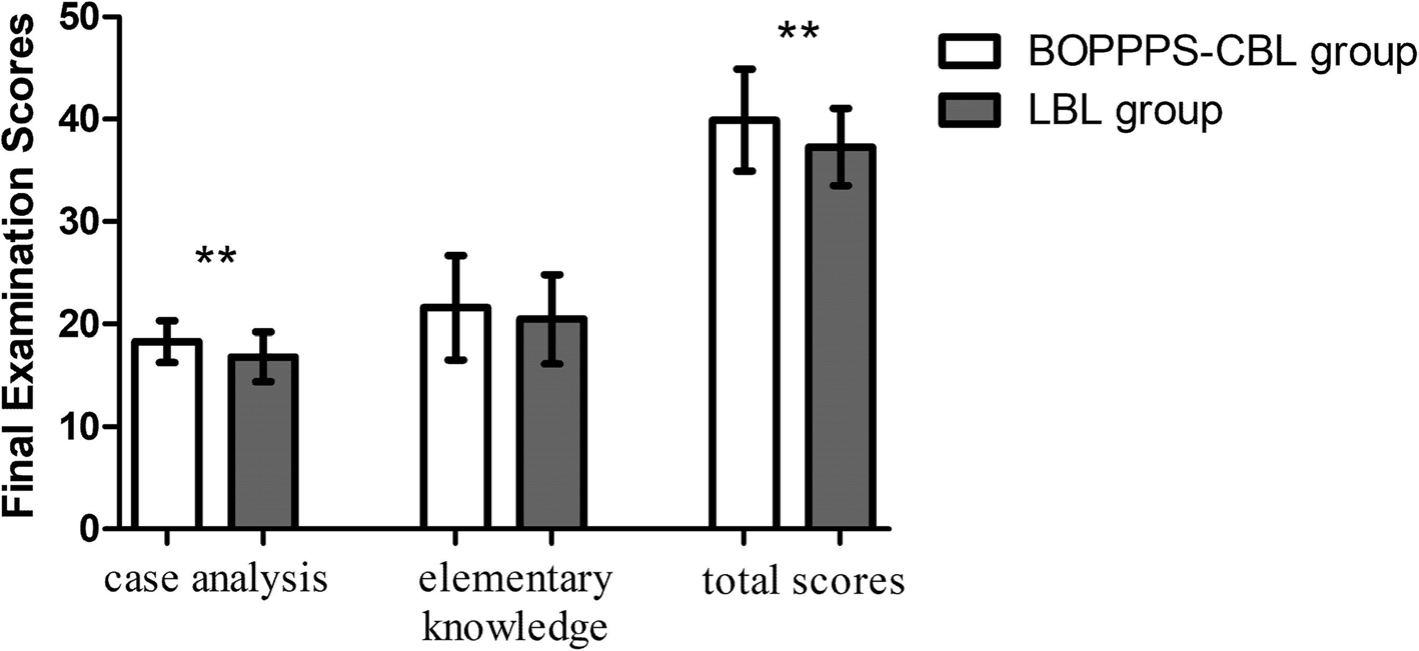
Comparison of the final examination scores between the BOPPPS-CBL group and the LBL group. ** indicates *P* < 0.01

The final examination scores of the BOPPPS-CBL group were significantly higher than those of the LBL group (39.91 ± 4.99 vs. 37.28 ± 3.77, *P* < 0.01), and the difference was statistically significant (*p* < 0.05). In terms of case analysis, the scores of the BOPPPS-CBL group were obviously higher than those of the LBL group (18.30 ± 2.02 vs. 16.81 ± 2.43, *P* < 0.01). There was no significant difference in fundamental knowledge scores between the two groups (21.60 ± 5.10 vs. 20.47 ± 4.33, *P* = 0.28) (Fig. 2).

## Discussion

Ophthalmology is a clinical subject with professional features. In recent decades, three themes evolving in ophthalmic medical education. Firstly, the focus has shifted from what to teach to how best to teach better. Secondly, medical education has evolved from teacher-centred to learner-centred. Lastly, medical education has shifted from an apprenticeship model to a new competency-based model of education [19]. However, there are still many problems in ophthalmology education during the process of teaching mode transformation. Ophthalmology falls into one of the smaller specialities and as such is allocated a smaller component of the total curriculum [20]. Academic experience and exposure to ophthalmology in medical school curricula have a global decline for decades [1, 4]. In addition, the COVID-19 pandemic has presented numerous challenges for ophthalmology education [21, 22]. There have been many novel and unique international educational innovations in ophthalmic curricula content (competency-based curriculum [20], structured academic curriculum [23]), teaching methodologies (virtual learning [21, 24], the online delivery of virtual ophthalmology clinic [25]), instructional design (team-based learning [26], flipped classrooms [16]) to enhance ophthalmology teaching to medical students education.

In China, paediatric ophthalmology is a subspecialty in ophthalmology and is a third-level subject. Ophthalmology education is a branch unit of sensory system teaching for five-year paediatric undergraduates at the College of Pediatrics of Chongqing Medical University. The traditional instructional approach focuses on LBL as the teaching centre, emphasizing the delivery of the syllabus and concepts [27]. Students passively accept the knowledge, leading to the reduction in learning initiative and enthusiasm. At present, traditional teaching methods cannot meet the needs of medical education students. New teaching methods are constantly being tested and improved by educators.CBL has been used in the medical field since at least 1912, when it was used by Dr. James Lorrain Smith while teaching pathology at the University of Edinburgh [28]. The goal of CBL is to prepare students for clinical practice through the use of authentic clinical cases. Unlike the traditional method, the CBL model links theory to practice through the application of knowledge to clinical cases using inquiry-based learning methods [17]. CBL requires advanced preparation by students and provides a more structural strategy for learning. It is based on concrete cases and characterized by effective and interactive teaching [17]. By discussing a clinical case related to the topic being taught, students evaluate their own understanding of the concept using a high order of cognition. This process encourages active learning and produces a more productive outcome [29].

The BOPPPS model, originating from North America, is a brand-new teaching model. The teaching process with a six-phase framework, including the bridge-in, objective, preassessment, participatory learning, post assessment and summary phases, emphasizes the participation of students through feedback during the teaching process [30]. In addition, this model accelerates the teaching cycle as a whole, including goals, behaviours, learning activities, and evaluation [31]. In recent years, the BOPPPS model, which pays more attention to the role of students’ initiatives in the teaching process and fully mobilizes their initiatives in the learning process, has been widely considered in China [32]. A large amount of the literature has shown the advantages of the BOPPPS model in various fields in health care and medicine [33], such as clinical medicine [34], dentistry [8], and histopathology [35].

In the current study, we applied the BOPPPS-CBL model to the ophthalmology teaching of five-year paediatric undergraduates. Compared with the traditional LBL teaching method, the BOPPPS-CBL model has several advantages originating from the above two teaching strategies. First, it is based on analyses of typical cases in ophthalmology. At the “bridge-in” stage, students can see the data and information for the cases, stimulating interest in learning. The “objective” stage makes students clearly understand the main content of this course in a framework. Our results showed that the students in the BOPPPS-CBL group knew more about the standard of work expected and had more motivation to learn.

Second, the BOPPPS approach changed the traditional relationships between teaching and learning. Teaching in the classroom is student-centred, with more emphasis on teacher-student interactions. In “participatory learning”, students purposefully acquire knowledge and link theory with practice based on the analysis of actual cases. The results of our survey showed that the BOPPPS-CBL model is more effective in developing students’ problem solving and analytical skills than the LBL model.

Finally, the final examination scores of the BOPPPS-CBL group were significantly higher than those of the LBL group, especially for case analysis. The score was not only an important and direct reference for evaluating the knowledge acquisition of the students but also an important parameter for measuring educational quality [14]. The results showed that the BOPPPS-CBL model led to better performance on the final test, but there were no significant differences in pressure between the two groups. In other words, the new teaching strategy did not increase the burden on students. Interestingly, more students in the LBL group thought the preclass workload was too high. A lack of real cases and boring theoretical information makes students feel more burdened. The bridge-in of real cases improved the students’ interest in learning, and the students in the BOPPPS-CBL group did not feel that the preclass workload was too high. By independently reviewing cases, discussing diagnoses and treatment suggestions, and offering advice to peers, the students consolidated their basic knowledge and benefitted from the model [14]. Therefore, the BOPPPS-CBL model is a very effective and acceptable method for ophthalmology education. The students’ perception scores indicated that the BOPPPS-CBL model was more beneficial to the development of problem-solving skills, analytical skills and motivation of learning.

### Limitations

This study has several limitations. First, the sample size was small. Research with additional samples is needed to validate the effect of this combined method. Second, the model was limited to a single unit of ophthalmology. The results may not be generalizable to students in other specialties. Third, the current study assessed only the final examination scores and lacked preclass and postclass tests to evaluate the learning effects. This may cause bias on learning performance. This study did not compare the differences between the CBL and BOPPPS models separately. A separate study could be designed to assess three separate groups CBL, BOPPPS and LBL in the future. In addition, the future study could perform a follow up exam at 1 year to determine the retention rate of the ophthalmic knowledge among the groups and to see whether there is a difference in long term retention for each teaching model.

## Conclusion

The current study indicates that the BOPPPS-CBL model is more efficacious than the traditional LBL model. The results of this study showed a significant increase in motivation and the effect of learning in the BOPPPS-CBL group. This model improves students’ enthusiasm for learning and helps cultivate their abilities to analyse and solve problems without increasing learning pressure. The BOPPPS-CBL model is an effective teaching model for ophthalmology education for five-year paediatric undergraduates. Further multicentre and large sample studies are warranted to verify whether CBL produces superior educational outcomes in other specialties and integrated teaching of the sensory systems.

## Data Availability

Our research involves in the student’s personal identity information. If the datasets analysed during the current study are publicly available, there would be a risk of revealing personal privacy. Therefore, the data is not publicly available. We declare that our data is not public. If you have a strong demand, please contact the corresponding author (L.Q 415712690@qq.com) to obtain.
